# USING HUMAN CENTERED DESIGN TO DEVELOP TWO NEW MEASURES OF LIVING WELL WITH DEMENTIA

**DOI:** 10.1093/geroni/igac059.2707

**Published:** 2022-12-20

**Authors:** Sheila Molony, Sam Fazio, Sheryl Zimmerman, Ricci Sanchez, Joe Montminy, Cindy Barrere, Rachel Montesano, Kimberly Van Haitsma

**Affiliations:** Quinnipiac University, Hamden, Connecticut, United States; Alzheimer’s Association, Chicago, Illinois, United States; University of North Carolina, Chapel Hill, North Carolina, United States; Alzheimer’s Association, Chicago, Illinois, United States; Alzheimer’s Association, Chicago, Illinois, United States; Quinnipiac University, Hamden, Connecticut, United States; Quinnipiac University, Hamden, Connecticut, United States; Pennsylvania State University, University Park, Pennsylvania, United States

## Abstract

**Background:**

New measures are needed that can provide a holistic approach to capturing salient outcomes contributing to quality of life in dementia. Purpose: To use human centered design to develop, prototype and evaluate new positive psychosocial measures for persons living with dementia

**Methods:**

This project used a conceptual framework of human centered design to obtain and analyze data from multiple sources, including individual and focus group interviews, observations in the field and related methods to enhance empathy for the lived experience of dementia, and to design prototypes for two new measures.

**Results:**

Qualitative analysis of interview data, integration of data from the theoretical and empirical literature and human centered design techniques including brainstorming and ideation were used to define constructs and create prototypes for a new Living Well with Dementia Inventory and Good Day-Bad Day assessment tool. Results of qualitative analysis, item development, and initial validity assessment will be presented.

**Conclusion:**

Human centered design is a useful method for development of person-centered measures. This method may complement traditional methods of instrument development and CBPR.

